# Screening a Phage Display Library for Two Novel OmpU-Binding Peptides with Adhesion Antagonistic Activity against *Vibrio mimicus*

**DOI:** 10.1371/journal.pone.0165092

**Published:** 2016-11-10

**Authors:** Lifang Qi, Yan Liu, Huizhu Tao, Ning Xiao, Jinnian Li, Lingyan Kong, Liting Hou

**Affiliations:** Key Laboratory of Zoonoses, College of Animal Science and Technology, Anhui Agricultural University, Hefei 230036, P. R. China; Imperial College London, UNITED KINGDOM

## Abstract

*Vibrio mimicus* is a pathogen that causes ascites disease in fish. We have previously demonstrated that the outer membrane protein U (OmpU) is an important adhesin in *V*. *mimicus*. Here eight specific OmpU-binding phage clones, which presented three different OmpU-binding peptides (designated P1, P2, P3), were screened from a commercially available phage displayed 12-mer peptide library using rOmpU protein as target. Then, synthetic OmpU-binding peptides were measured for their adhesion antagonistic activity and binding affinity via adhesion inhibition test and non-competitive ELISA, respectively. The results showed that after co-incubated with the mixture of rOmpU and P3, visible green fluorescence could be observed on the *epithelioma papulosum cyprinidi* (EPC) cells surface; while the EPC cells co-incubated with the mixture of rOmpU and P1/P2 exhibited little green fluorescence. The average adhesion number of *V*. *mimicus* 04–14 isolate before and after treatment with peptide was 21.4 ± 1.5, 20.8 ± 0.8 (irrelevant peptide), 20.2 ± 0.5 (P3), 5.1 ± 0.7 (P1) and 3.4 ± 0.8 (P2), respectively. There was a significant decrease in the adhesive level of 04–14 isolate treated with P1/ P2 compared to the untreated isolate (*p*<0.01). The affinity constants of P1 and P2 were (6.17 ± 0.19) × 10^8^ L/mol and (1.24 ± 0.56) × 10^9^ L/mol, respectively. Furthermore, protective effects of P1 and P2 on grass carps challenged with *V*. *mimicus* were preliminary detected. It was found there was delayed death of fish in the groups treated with P1/P2, and the survival rate of challenged fish improved with the increase of the dose of adhesion antagonistic peptide. Taken together, two novel OmpU-binding peptides, which possessed adhesion antagonistic activity, high affinity and a certain degree of antibacterial activity against *V*. *mimicus*, were screened and identified.

## Introduction

*Vibrio mimicus* (*V*. *mimicus*) is a common pathogen found in aquaculture that can cause severe ascites disease in fish [1–2]. The controlling methods for ascites disease in fish at present are still mainly oral antibiotics and topical chemical disinfectants. However, the frequent use of antibiotics not only causes pollution of the water environment and lower quality of aquatic products, but also induces more and more drug resistant strains, which led to the death of many fish and economic losses to the aquaculture industry. Thus, the development of novel antimicrobial agents to prevent or treat ascites disease in fish is vitally important for the aquaculture industry.

Bioactive peptides can exert biological effects by highly selective binding to active site on a target protein *in vitro* and *in vivo*. Moreover, they have several advantages, such as lower manufacturing costs, higher activity per unit mass, generally lower immunogenicity, little toxicity and better organ penetration. Therefore, bioactive peptides are receiving attention as alternatives to antibiotics [3–4]. In previous studies, we have demonstrated that *V*. *mimicus* is an enteric pathogen and the outer membrane protein U (OmpU) is an important adhesin in *V*. *mimicus*. The ability of *V*. *mimicus* to adhere and colonize the intestinal mucosal cells via OmpU adhesin is the key step that initiates ascites disease in fish [5–6]. Creating adhesion antagonist that can block or antagonize *V*. *mimicus* specifically adhesion to cells is a potential new strategy for the prevention and treatment of ascites disease in fish.

Phage display is a molecular technology that allows the presentation of a large number of peptides on the phage surface. The display of peptide libraries on the surface of bacteriophage permits the selection of peptides with high affinity and specificity for almost any target by biopanning. At present, the technology has been widely used in epitope screening, new vaccine development, peptide drug design and so on [7–9]. The purpose of this study was to screen OmpU-binding peptides by scanning a commercially available phage displayed 12-mer peptide library with purified rOmpU fusion protein. Then, the adhesion antagonistic activity and binding affinity of OmpU-binding peptides were determined via adhesion inhibition test and solid phase non-competitive ELISA, respectively. Furthermore, their protective effects on grass carps (*Ctenopharyngodon idella*) challenged with *V*. *mimicus* was preliminarily detected. Our findings may provide a basis to develop adhesion antagonistic peptide against *V*. *mimicus* infection.

## Materials and Methods

### Ethics statements

The bacterial strain used in this study was isolated in our previous studies [6]. *Epithelioma papulosum cyprini* (EPC) cell line was obtained from the Ministry of Agriculture Fisheries Pathogenic Library. Rabbit anti-OmpU antibody and the preimmune serum were available from previous studies [10], and other materials were bought from legal biotechnology companies.

All animal experiments were carried out in strict accordance with the recommendations in the Guide for the Care and Use of Laboratory Animals of the national laboratory animal welfare ethics, and protocols concerning animals were approved by the Ethical Committee of the Faculty of Veterinary Science of Anhui Agricultural University (Permit Number: 20130402). Every effort was made to reduce the number of animals used and minimize the suffering of the animals.

### Bacterial strains and the phage peptide library

The *V*. *mimicus* 04–14 isolate was obtained from *Ctenopharyngodon idella* with ascites disease and then identified using the API 20 NE system and 16S rRNA gene sequencing as in our earlier studies [6]. The isolate was cultured in brain heart infusion broth (BHI; Beijing Solarbio Science & Technology Co., Ltd., China) at 30°C. The Ph.D.-12^TM^ Phage Display Peptide Library Kit containing *E*. *coli* host strain ER2738 and _96 gIII sequencing primer was purchased from New England BioLabs.

### Cell line and cell culture

EPC cell line was obtained from the Ministry of Agriculture Fisheries Pathogenic Library. EPC cells were maintained in M199 medium (Gibco Life Technologies) supplemented with conventional antibiotics and 10% fetal bovine serum (Gibco Life Technologies). The cells were cultured at 28°C in a 5% CO_2_ atmosphere and were trypsinized and separated into fresh medium at a volume ratio of 1:4 at least once a week.

### Biopanning of phage random peptide library against recombinant OmpU fusion protein

Biopanning was performed according to the instructions of Ph.D.-12^TM^ Phage Display Peptide Library Kit. Briefly, 96-well microplates (Corning, USA) were coated with purified rOmpU fusion protein and His-tag protein (prepared in our previous test [10]), 10 μg/well, respectively. The plates were incubated at 4°C overnight, and wells were blocked with 3% bovine serum albumin (BSA; Sigma) at 37°C for 2 h followed by six washes with TBST (TBS plus 0.1% Tween-20). Next, the diluted phage peptide library was added to His tag protein-coated wells, 1 × 10^11^ PFU/well, and the plates were incubated at room temperature for 1 h with gentle shaking. The phage solution unbound with the His-tag protein was aspirated and used as the pre-screening solution. The pre-screening solution was added to rOmpU fusion protein coated wells, 100 μL/well, and the plates were incubated at room temperature for 1 h with gentle shaking. Unbound phages were discarded from the wells by washing 6 times with TBST. Any bound phage was eluted with elution buffer (Glycine-HCl 0.2 mol/L, pH = 2.2 and BSA 1 mg/mL). The eluted phages were amplified in *E*. *coli* ER2537 and concentrated by polyethylene glycol precipitation. For each subsequent round of panning, the input number of phages was the same as the first round. After 4 rounds of screening, phage clones binding to rOmpU fusion protein could be effectively enriched by gradually reducing the rOmpU concentration from 100 μg/mL to 10 μg/mL and gradually increasing the Tween-20 concentration in the washing solution from 0.1% to 0.5%.

### ELISA identification of positive phage clones

To obtain positive phage clones that specifically bind to OmpU protein, after four rounds of biopanning, a total of 15 individual phage clones were randomly selected for initial identification by phage ELISA-binding assay. Briefly, 96-well plates were coated with purified rOmpU fusion protein (10 μg/well) overnight at 4°C. The plates were washed three times with PBST and blocked with 5% BSA for 2 h at 37°C. Previously selected phage clones were amplified and titred according to the phage libraries manufacturer′s instructions. The diluted phage clones were then added to rOmpU-coated wells (1 × 10^10^ PFU/well), and the plates were incubated at 37°C for 2 h. After 3 washes in TBST, bound phages were subjected to reaction with horseradish peroxidase (HRP)-conjugated anti-M13 monoclonal antibody (Pharmacia, USA) at 1:5,000 dilution in PBS at 37°C for 1 h, followed by color development with o-phenylenediamine dihydrochloride (OPD) substrate solution, and the OD_492nm_ value was measured in a microplate Reader. In the test, M13 phage in peptide library was used as negative control and TBST as blank control. Each clone experiment was performed in triplicate. If the mean OD_492nm_ value of a tested phage clone was 3 times greater than the mean OD_492nm_ value of negative control, then the phage clone was preliminarily identified as positive.

Competitive inhibiting ELISA was used to further identify preliminary positive phage clones. Briefly, 96-well plates were coated with purified rOmpU and blocked as described above. Different concentrations of free rOmpU (6.25 μg/mL, 12.5 μg/mL, 25 μg/mL, 50 μg/mL, 100 μg/mL, 200 μg/mL) were mixed with an equal volume of preliminary positive phage clones (final concentration 1 × 10^10^ PFU/well) and incubated at 37°C for 30 min followed by adding to rOmpU-coated wells (100 μL/well), and the plates were incubated at 37°C for 1h. The remaining steps were the same as for " phage ELISA-binding assay " (*vide supra*). At the same time, the uninhibition control (PBS instead of competitor mixed with preliminary positive phage clones at equal volume) was also analyzed. The results were expressed as inhibition ratio that was calculated with the following formula: (OD_492nm_ value unsuppressed—OD_492nm_ value after suppression) / OD_492nm_ value unsuppressed × 100%. This assay was performed in triplicate.

### DNA sequencing of the positive phage clones and synthesis of OmpU-binding peptides

Single-strand DNA from ELISA-positive phage clones was extracted as described in the DNA extraction kit (Omega, USA) and sequenced by Shanghai Biological Engineering Technology Co. Ltd with the _96gIII sequencing primer. The amino acid sequence deduced from the inserted nucleotide sequences were analyzed by the DNASTAR Lasergene program.

To measure their activity and affinity constant, OmpU-binding peptide and an irrelevant control peptide (i.e., a partial sequence of OmpU protein signal peptide) were synthesized at the Shanghai Amoy Cape Technology Co., Ltd. The peptide purity was verified by HPLC-analysis and estimated ≥ 98%.

### Effects of OmpU-binding peptides on OmpU protein adhesion to EPC cells

Synthetic OmpU-binding peptides were measure for their ability to antagonize OmpU protein adhesion to EPC cells by fluorescence-based adhesion assay. Briefly, EPC cells (1 × 10^6^ cells/mL) were seeded in 24-well cell plate containing coverslip (Corning, NY, USA) and cultured to 80% confluence. The cells were gently washed 3 times with PBS and fixed with 4% paraformaldehyde at room temperature for 15 min. After 3 washes with PBS, cells were permeabilized with 0.5% Triton X-100 (500 μL/well) for 15 min. The cells were then washed 3 times with PBS and blocked with 2% BSA for 30 min. Subsequently, 500 μg/mL of each OmpU-binding peptide or irrelevant control peptide (used as negative control) solution were respectively mixed with an equal volume of 500 μg/mL rOmpU fusion protein, and the mixture was incubated for 1 h at 28°C and added to each well. The plates were incubated for 1 h at 28°C. After 3 washes, purified rabbit anti-OmpU antibody (prepared in our previous studies [10]) at a dilution of 1:200 in PBS was added to each well and incubated for 1 h at 28°C, followed by 3 washes in PBS. A 1:100 dilution of FITC (fluorescein isothiocyanate)-labeled goat anti-rabbit IgG (Jackson ImmunoResearch Laboratories, Inc., USA) was added to each well and incubated in the dark for 1 h. After 3 washes in PBS, 4,6-Diamidino-2-phenylindole dihydrochloride (DAPI; Ziyi Reagent Co., Shanghai, China) was used to stain the nucleus, and the coverslips were observed under a fluorescence microscope. Each experiment was performed in triplicate, each sample being tested in three wells.

### Effects of OmpU-binding peptides on *V*. *mimicus* adhesion to EPC cells

When EPC cells grown in 24-well plate containing coverslips formed a confluent monolayer, the medium was changed to RPMI-1640 medium without serum and antibiotics for 2 h. Subsequently, 500 μg/mL of each OmpU-binding peptide or unrelated control peptide solution were respectively mixed with an equal volume of *V*. *mimicus* isolate 04–14 suspension (2 × 10^7^ CFU/mL) and incubated for 1 h at 28°C. The mixture was added to each well, and then the plates were incubated for 1 h at 28°C. After 3 times washes, EPC cells were fixed with cooled methanol for 10 min, stained with crystal violet and examined under a light microscope at a magnification of ×1,000. Thirty cells were randomly selected to count the number of adhering bacterial per slide. Each experiment was performed in triplicate, with each sample being repeated in 3 cell wells.

### Affinity constant determination of the adhesion antagonistic peptides

To determine the binding affinity of interactions between adhesion antagonistic peptide and rOmpU fusion protein, solid phase non-competitive ELISA method (also known as the Beatty method [11]) was used to measure the affinity constant of adhesion antagonistic peptide. Briefly, adhesion antagonistic peptides were diluted to 40 μg/mL, 20 μg/mL, 10 μg/mL, 5 μg/mL and 2.5 μg/mL, respectively, and laterally coated onto plate wells (100 μL/well) at 4°C overnight. Next day, each well was blocked with 5% BSA at 37°C for 2 h, and then washed 6 times with TBST. Subsequently, rOmpU fusion protein were diluted to 12.8 μg/mL, 3.2 μg/mL, 0.8 μg/mL and 0.2 μg/mL, respectively, and longitudinally added to coated wells (100 μL/well), and the plates were incubated for 2 h at 37°C. After thorough washing, rabbit anti-rOmpU protein antibody, HRP labeled goat anti-rabbit IgG and substrate solution was added in turn, each step was preceded by 6 washes in TBST. OD_492_ nm values were finally determined. With OD_492_nm values as the ordinate and adhesion antagonistic peptide concentration as the abscissa, the binding reaction curves between adhesion antagonistic peptide and rOmpU fusion protein at different concentrations were plotted. MATLAB version 7.0 was used to fitting the binding response curve, and to derive the curve equation. From the equation, the corresponding adhesion antagonistic peptide concentration at 50% OD_492_nm value in the curve was obtained. The affinity constant K was calculated using a modified version of the Beatty formula [affinity constant (K) = (n—1) / 2 (nAb'_total_- Ab_total_)] [11]. Ab'_total_ and Ab_total_ in the formula represent respectively the corresponding adhesion antagonistic peptide concentration at 50% OD_492_nm value in the binding response curve when the concentration of rOmpU protein was P' and P; n represents P/P'. This assay was performed in triplicate.

### Acute toxicity detection of the adhesion antagonistic peptides to grass carps

Healthy grass carps weighting 50–52 g were purchased from a commercial fish farm in Anhui province of China, and then were maintained in a flow-through water system at a temperature of 25 ± 1°C and fed commercial pellets on a daily basis. After 14 days when grass carps were confirmed to be healthy, the water temperature was increased to 28 ± 1°C slowly, and the experiment began.

For acute toxicity detection, synthetic P1, P2 and unrelated control peptide was dissolved in sterile deionized water, respectively, and given to grass carps by intraperitoneal (i.p.) injection at a dose of 100 mg/kg, six fish per group. The number of fish surviving in each group was monitored for up to a period of 7 days after injection. At the end of the experiments, all surviving fish were euthanized in a solution of 200  mg/L of MS-222 followed by a blow to the head.

### Preliminary detection of protective effects of the adhesion antagonistic peptides on grass carps challenged with *V*. *mimicus*

The experimental grass carps were randomly divided into 9 groups (named A-I group, n = 10 in each group). Groups A-C were injected intraperitoneally with P1 at a dose of 10 mg/kg/d, 20 mg/kg/d and 30 mg/kg/d, respectively; Groups D-F were given the same dosages of P2, respectively; Group G was given with 30 mg/kg/d of unrelated control peptide. After 3 days of continuous peptides injection, each fish in groups A-G was challenged intraperitoneally with 200 μL PBS containing 6 × 10^6^ cells of *V*.*mimicus* 04–14 isolate (equal to the minimal lethal dose determined by preliminary test), respectively. Group H (infection control group) was only inoculated with 04–14 isolate at the minimal lethal dose; Group I (negative control group) was only inoculated with 0.65% sterile saline. Survival of the fish was monitored every 12 h for 7 days post-challenge. At the end of the 7 day observation period, all surviving fish were euthanized in a solution of 200  mg/L of MS-222 followed by a blow to the head.

The survival experiment was performed in accordance with the practices reported in recent studies on antibacterial efficacy of peptides *in vivo*, protective efficacy of vaccine and bacterial virulence [12–14]. Death was used as an endpoint in these studies (with no indication of use of humane euthanasia). In animal toxicity and efficacy assays of the adhesion antagonistic peptides, we used death as the endpoint as early, reliable and unbiased biomarkers that can be used as indicators of disease severity are lacking. There is difficulty in reliably differentiating fish that will die from those which will recover, despite showing clinical signs precluding use of humane euthanasia. In addition, interpretation of clinical signs can itself be subjective in the absence of quantifiable indicators of disease severity. For these reasons, the ethical committee of Anhui Agricultural University has assessed the possibility of deaths in the animal toxicity and efficacy assays and also allowed probably unavoidable animal deaths.

### Statistical analysis

Data were presented as mean ± standard deviations (SD) from three independent experiments. All statistical analyses were performed using SAS for Windows version 9.0 (SAS, Chicago, USA). Student’s t test was used to compare data in two groups, and one-way ANOVA followed by Tukey’smultiple comparison test was used for multiple comparison data. A *p* value of <0.05 was considered as statistically significant.

## Results

### Selection of OmpU-binding phage clones and analysis of peptide sequences

**Fig 1:**
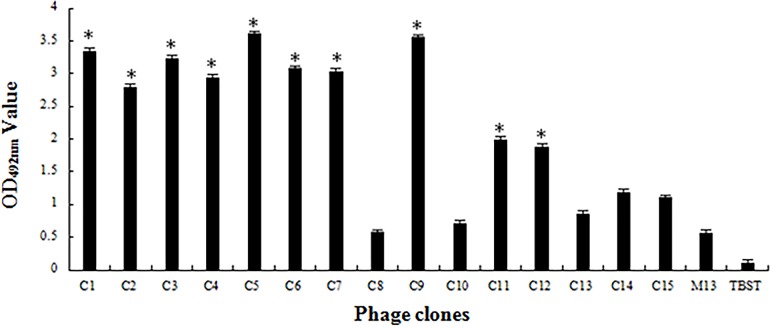
Phage ELISA OmpU-binding assay. Purified rOmpU fusion protein was served as coated antigen, bound phage was detected with HRP-conjugated anti-M13 antibody. Wild-type M13 phage was served as negative control. Data presented are the mean OD_490nm_ values (±SD) of triplicate samples, and the error bars indicate the standard deviations from the means. The asterisk represents the preliminary positive clones whose OD_490nm_ values are three times greater than that of M13 phage.

**Fig 2:**
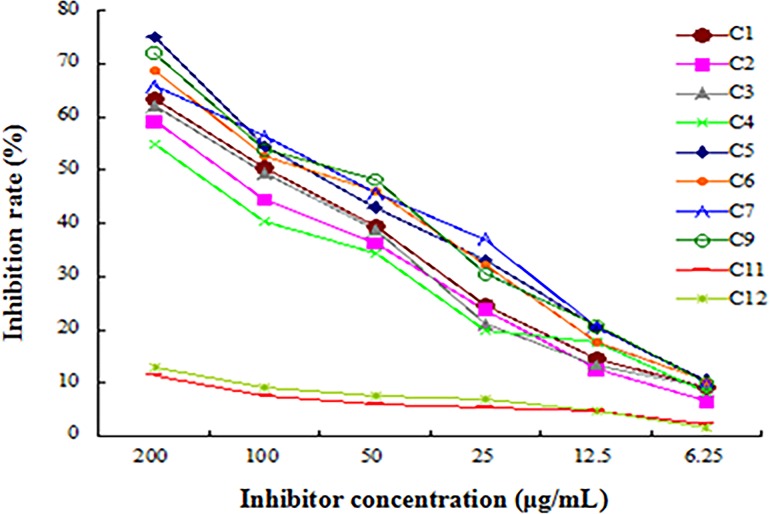
Competitive inhibition of preliminary positive phage clones to coated rOmpU fusion protein in the presence of free rOmpU competitor. The average inhibition rates (means ± SE, n = 3) at different concentrations of free rOmpU fusion protein were shown. The binding of preliminary positive phage clone1/2/3/4/5/6/7/9 to coated rOmpU protein was significantly inhibited by free rOmpU inhibitor in a concentration-dependent manner.

After four rounds of biopanning, 15 phage clones were selected randomly for detection. 10 phage clones were preliminarily identified as positive by phage ELISA-binding assay (Fig 1) and were further identified by competitive inhibition ELISA. As shown in Fig 2, after free rOmpU fusion protein co-incubated with 10 preliminary positive phage clones, respectively, free rOmpU fusion protein competitively inhibited 8 phage clones binding to coated rOmpU in a concentration-dependent manner. The results revealed that these clones (C1, C2, C3, C4, C5, C6, C7, C9) were positive phage clones. The DNA inserts of the positive phage clones were sequenced, and their amino acid sequences were deduced. As shown in Table 1, three different OmpU-binding peptide sequences were obtained, in which the peptide sequence displayed on clones C1/C2/C3 was identical (named P1) while C5/C6/C7/C9 displayed peptide sequence was identical (named P2). The C4 clone exhibited another different peptide sequence that was termed P3.

**Table 1:** OmpU-binding peptide sequences obtained by screening phage random 12-peptide library against rOmpU fusion protein.

*OmpU-binding peptide*		*Phage clones*	*Amino acid sequence*	*Frequency*
P1	C1/C2/C3		TWTCSDVICTAR	3
P2	C5/C6/C7/C9		SCTSPHCFMWLP	4
P3	C4		TGYICDEMFCKL	1

### Adhesion antagonistic activity of the OmpU-binding peptides

**Fig 3:**
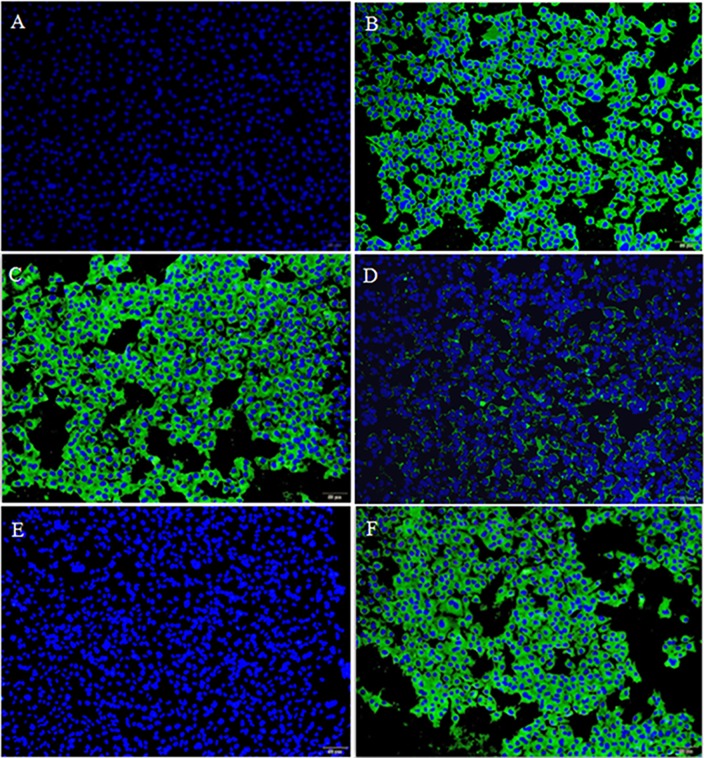
Inhibition of rOmpU fusion protein to EPC cells in the presence of OmpU-binding peptide determined by fluorescence-based adhesion assay. **(A)** EPC cells were co-incubated with M199 medium. Nuclei was stained with DAPI (blue color). **(B)** EPC cells were co-incubated with rOmpU fusion protein, followed by washing and visualization of surface retained rOmpU by immunofluorescent staining with OmpU-specific antibodies (green color). Nuclei was counterstained with DAPI (blue color). (C) EPC cells were co-incubated in the mixture of rOmpU with an irrelevant control peptide, same staining as in (B). **(D), (E) and (F)** EPC cells were co-incubated in the mixture of rOmpU with an irrelevant control peptide or OmpU-binding peptide1,2,3, respectively, same staining as in (B). All scale bars are 100μm.

Fluorescence-based adhesion assay was used to detect whether OmpU-binding peptides had adhesion antagonistic activity against rOmpU fusion protein binding to EPC cells. As shown in Fig 3, the surface of EPC cells co-incubated in the mixture containing the rOmpU fusion protein and P1 or P2 exhibited little or no green fluorescence (Fig 3D and 3E), the nucleus being stained blue in all experimental groups. Obvious green fluorescence could be observed on the surface of EPC cells co-incubated in the mixture containing rOmpU fusion protein and an unrelated control peptide or P3 (Fig 3C and 3F). Negative control cells co-incubation with M199 medium did not emit any green signal (Fig 3A). Positive control cells co-incubation with rOmpU fusion protein exhibited clear green fluorescence (Fig 3B).

**Fig 4:**
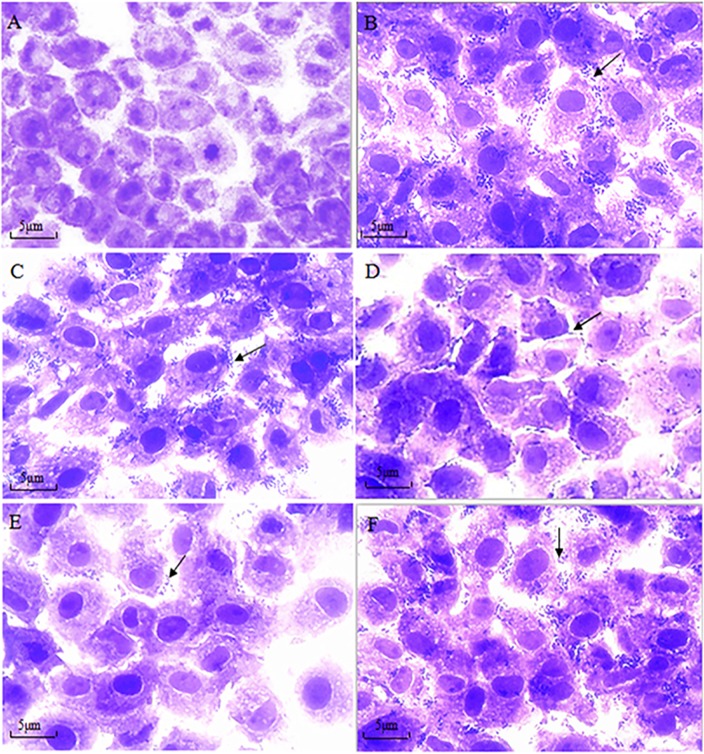
Inhibition of *V*.*mimicus* adhesion to EPC cells in the presence of OmpU-binding peptide determined by adhesion inhibition assay. **(A)** Micrographs of control EPC cells stained with crystal violet; **(B)** Adhesion of *V*.*mimicus* 04–14 isolate to EPC cells, same staining as in (A). **(C)** Adhesion of 04–14 isolate to EPC cells after treatment with an irrelevant peptide same staining as in (A). **(D), (E) and (F)** Adhesion of 04–14 isolate to EPC cells after treatment with OmpU-binding peptide 1, 2, 3, respectively, same staining as in (A). Magnification 1000 ×. All scale bars are 5μm. Arrows show *V*. *mimicus* 04–14 isolate.

A peptide adhesion inhibition test was used to further examine the effect of OmpU-binding peptide on *V*. *mimicus* adhesion to EPC cells. As shown in Fig 4, control EPC cells showed neither bacterial adhesion nor cytopathic effects (Fig 4A). *V*. *mimicus* 04–14 isolate alone and after treatment with a irrelevant control peptide or P3 could adhere to cells in an aggregative manner, the average number of adhesion around each cell being 21.4 ± 1.5, 20.8 ± 0.8 and 20.2 ± 0.5, respectively (Fig 4B, 4C and 4F). The adhesion of 04–14 isolate treated with P1 or P2 to EPC cells was significantly decreased, with the average number of adhesion being 5.1 ± 0.6 and 3.4 ± 0.8, respectively (*p*<0.05) (Fig 4D and 4E). The results of two separate tests above reveal that among the three OmpU-binding peptides, P1 and P2 possessed adhesion antagonistic activity.

### Binding affinity of interactions between the adhesion antagonistic peptide and rOmpU fusion protein

**Fig 5:**
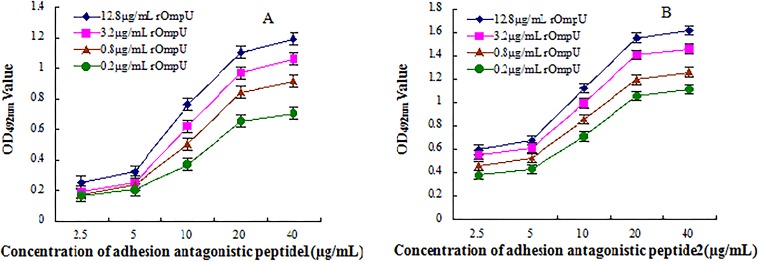
Binding reaction curve of adhesion antagonistic peptide—rOmpU protein interactions determined by solid phase non-competitive ELISA method. **(A)** Binding reaction curve between P1 (from 2.5 to 40 μg/mL) and rOmpU fusion protein (from 0.2 to 12.8 μg/mL) at four different concentrations, with OD_492_nm values as the ordinate and adhesion antagonistic peptide concentration as the abscissa. **(B)** Binding reaction curve between P2 and rOmpU fusion protein at the same concentrations as in (A).

The interactions reaction curve between adhesion antagonistic peptide and rOmpU fusion protein was shown in Fig 5. The curve equation was deduced using MATLAB software. From the curve equation, the corresponding adhesion antagonistic peptide concentration was calculated at the 50% OD_492_nm value in the curve (Table 2), and then entered into the Beatty equation. The affinity constants of adhesion antagonistic peptides P1 and P2 were (6.17 ± 0.19) × 10^8^ L/mol and (1.24 ± 0.56) × 10^9^ L/mol, respectively. The binding affinity of P2 was significantly stronger than that of P1 (*p*<0.05).

**Table 2:** Deduced binding reaction curve equation of adhesion antagonistic peptide—rOmpU fusion protein and the corresponding peptide concentration at 50% OD_492nm_ value in the curve.

*rOmpU concentration(μg/mL)*	*Curve Equation^*b*^*		*AAP^a^ concentration at 50% OD_492nm_ (μg/mL)*		
	AAP1	AAP2	AAP1		AAP2
0.2	y = 0.7/[1+3.4exp(-1.1x)]	y = 1.1/[1+2.2exp(-1.1x)]		1.12	0.70
0.8	y = 0.9/[1+4.7exp(-1.5x)]	y = 1.2/[1+2.0exp(-1.2x)]		1.05	0.57
3.2	y = 1.0/[1+5.3exp(-1.7x)]	y = 1.4/[1+1.9exp(-1.2x)]		0.98	0.56
12.8	y = 1.2/[1+4.5exp(-1.8x)]	y = 1.6/[1+2.0exp(-1.3x)]		0.86	0.55

^**a**^AAP represents adhesion antagonistic peptide.

^b^The x in curve equation indicates concentration of adhesion antagonistic peptide, y indicates OD_492_nm value.

### Acute toxicity detection of the adhesion antagonistic peptides to grass carps

The grass carps injected with the dose of 100 mg/kg adhesion antagonistic peptide (P1, P2) and a unrelated control peptide by i.p showed no immediate adverse events, such as enervation or feed intake reduction, and all the treated fish survived in the 7-day study period, suggesting that these peptides themselves were not acute toxicity to grass carps.

### Protective effects of the adhesion antagonistic peptides on grass carps

As shown in Table 3, grass carps in the non-infected control group were healthy without death. All fish in the infected control group and irrelevant peptide control group died within 48 h after challenge. There was delayed death of the grass carps in the groups treated with adhesion antagonistic peptides, and the survival rate of challenged fish improved with the dose increase of adhesion antagonistic peptide. At the dose of 30 mg/kg/d, the survival rate of challenged grass carps in groups P1 and P2 reached 40% and 60% in the 7-day study period, respectively. These data indicate preliminarily that P1 and P2 possessed some protective effects on grass carps challenged with *V*. *mimicus*.

**Table 3:** Protective effects of the adhesion antagonistic peptides on grass carps challenged with *V*. *mimicus*.

*Group*^*a*^	*No*. *of dead fish at different times post challenge*							*No*. *of dead fish*	*Mortality rate (%)*	*Survival rate (%)*
	1d	2d	3d	4d	5d	6d	7d			
A	0	5	2	2	0	0	0	9	90	10
B	0	4	2	2	0	0	0	8	80	20
C	0	2	3	1	0	0	0	6	60	40
D	0	4	3	1	0	0	0	8	80	20
E	0	2	3	1	0	0	0	6	60	40
F	0	2	2	0	0	0	0	4	40	60
G	4	6	0	0	0	0	0	10	100	0
H	7	3	0	0	0	0	0	10	100	0
I	0	0	0	0	0	0	0	0	0	100

^a^A-C. injected with P1 at a dose of 10 mg/kg/d,20 mg/kg/d and 30 mg/kg/d, respectively, prior to challenge; D-F. injected with P2 at the dose of 10 mg/kg/d,20 mg/kg/d and 30 mg/kg/d, respectively, prior to challenge; G. injected with 30 mg/kg/d dose of an irrelevant control peptide prior to challenge; H. infection control group; I. non-infection control group.

## Discussion

Phage display technology has been found to be an excellent tool in successfully identifying affinity peptides against a variety of targets [15–16]. In present study, recombinant His-OmpU fusion protein was used as a target to screen phage displayed 12-mer peptide library. Because of the huge diversity of the phage random 12-peptide library and the His-tag protein interference, there may be a possibility that a variety of peptides bound to the different sites of the target were obtained [17]. In order to absorb non-specificity phage clones and obtain specific phage clones with high affinity, we first used His-tag protein to pre-screen original phage library before screening with rOmpU fusion protein, then processed the selection round by round via decreasing rOmpU fusion protein concentration, shortening the binding time of rOmpU fusion protein with peptide library, and increasing Tween-20 concentration in the eluent to reduce the enrichment of non-specific phage clones. After 4 rounds of biopanning, 10 of 15 phage clones picked randomly were preliminarily identified as positive by phage ELISA-binding assay. However, it was unclear if these clones bound to the OmpU protein or His-tag protein. Hence, specific interactions between the preliminary positive phage clone and OmpU protein were further analyzed by competitive inhibition ELISA. In this specific test, 8 of the suspect 10 clones were proved to be positive, which presented 3 different OmpU-binding peptide sequences, indicating this pre-screening approach is feasible.

The two peptides (P1 and P2) of 3 OmpU-binding peptides were identified as adhesion antagonistic peptide by adhesion inhibition test, which could inhibit the specific adhesion of *V*. *mimicus* and OmpU protein to EPC cells. Notably, preliminary experiment *in vivo* showed that three successive pre-treatment with P1 or P2 just before *V*. *mimicus* exposure could provide some protection using fish death as an end point. We speculate that these peptides may play an antibacterial efficacy *in vivo* through inhibition of the adhesion of *V*. *mimicus* OmpU protein to susceptible cells in fish, although the target molecules on fish cells where OmpU binds are not yet clear. Usually, bioactive peptides and proteins exhibit short plasma half-life resulting from enzymatic degradation by protease and rapid clearance by renal filtration in systematic circulation. This feature hampers their application in clinical prevention and treatment. In order to improve adhesion antagonistic activity of two selected peptides and extend their the half-life *in vivo*, we will design a tandemly arranged adhesion antagonistic peptide with two selected peptides linked with two alanines (A) and a tyrosine (Y) as an AAY spacer, then conjugate the tandemly arranged adhesion antagonistic peptide to albumin to construct fusion protein via genetic engineering technology and evaluate on their adhesion antagonistic activity, half-life and long-term toxicity to growth and reproduction of fish.

Whether adhesion antagonistic peptides can effectively inhibit the bacterial adhesion depends on the binding affinity between the antagonistic peptide and adhesin. The stronger the affinity is, the higher adhesion antagonistic activity will be. The strength of the affinity is represented by the affinity constant. Equilibrium dialysis is the classical method used to determine the affinity constant, but it is only suitable for small molecule antigen and antibody, and the operations involved are quite complicated [18]. The solid phase non-competitive ELISA method for the determination of affinity constants is not limited by the antigen size and it is easy to operate, but some scholars believed that antigen-antibody reaction kinetics would be changed and the application of the Law of Mass Action would be limited, because the antigen-antibody reaction is transferred from a liquid phase to the solid-liquid interface, caused by the antigen has been coated in microtitre plate [19]. To solve this problem, Beatty and Loomans [11, 20] used non-competitive ELISA and equilibrium dialysis methods to determine the affinity constant of anti-CEA 1gG at the same time, and found that the affinity obtained from two methods was highly consistent. Hence, they confirmed that in strictly solid phase ELISA-controlled conditions, the Law of Mass Action is still applied and the results are reliable. At present, solid phase non-competitive ELISA has been used to determine the affinity not only between antigen and antibody, but also between polypeptide molecules and target protein [21–24]. In present study, the affinity constants of both adhesion antagonistic peptides were measured by the method. The data showed that the binding affinity of P2- rOmpU was significantly stronger than that of P1- rOmpU (*p*<0.05), which paralleled its higher adhesion antagonistic activity *in vitro* and antibacterial efficacy *in vivo*.

## Conclusion

This is the first report to uncovered two novel OmpU-binding peptides with adhesion antagonistic activity, high affinity and a certain degree of antibacterial activity against *V*. *mimicus*. Both peptides may have potential applications in the development for adhesion antagonists against *V*. *mimicus* infection.
